# The Influence of the Amplitude of Low-Frequency Fluctuations on Resting-State Functional Connectivity

**DOI:** 10.3389/fnhum.2013.00118

**Published:** 2013-04-02

**Authors:** Xin Di, Eun H. Kim, Chu-Chung Huang, Shih-Jen Tsai, Ching-Po Lin, Bharat B. Biswal

**Affiliations:** ^1^Department of Biomedical Engineering, New Jersey Institute of TechnologyNewark, NJ, USA; ^2^Institute of Neuroscience, National Yang-Ming UniversityTaipei, Taiwan

**Keywords:** ALFF, basal ganglia, brain network, default mode network, independent component analysis, insula, thalamus

## Abstract

Studies of brain functional connectivity have provided a better understanding of organization and integration of large-scale brain networks. Functional connectivity using resting-state functional magnetic resonance imaging (fMRI) is typically based upon the correlations of the low-frequency fluctuation of fMRI signals. Reproducible spatial maps in the brain have also been observed using the amplitude of low-frequency fluctuations (ALFF) in resting-state. However, little is known about the influence of the ALFF on the functional connectivity measures. In the present study, we analyzed resting-state fMRI data on 79 healthy old individuals. Spatial independent component analysis and regions of interest (ROIs) based connectivity analysis were performed to obtain measures of functional connectivity. ALFF maps were also calculated. First, voxel-matched inter-subject correlations were computed between back-reconstructed IC and ALFF maps. For all the resting-state networks, there was a consistent correlation between ALFF variability and network strengths (within regions that had high IC strengths). Next, inter-subject variance of correlations across 160 functionally defined ROIs were correlated with the corresponding ALFF variance. The connectivity of several ROIs to other regions were more likely to correlate with its own regional ALFF. These regions were mainly located in the anterior cingulate cortex, medial prefrontal cortex, precuneus, insula, basal ganglia, and thalamus. These associations may suggest a functional significance of functional connectivity modulations. Alternatively, the fluctuation amplitudes may arise from physiological noises, and therefore, need to be controlled when studying resting-state functional connectivity.

## Introduction

Studies of brain networks and functional connectivity have provided a better understanding of organization and integration of large-scale brain networks. After the initial observation that the motor cortex exhibits highly synchronized intrinsic fluctuations during the absence of specific tasks (Biswal et al., 1995), the resting-state functional connectivity has emerged as a promising approach to investigate the functional integration of the brain. Studies using seed-based correlations have shown that the resting-state BOLD signal of functionally related regions generally demonstrate high correlation coefficients (e.g., Cordes et al., 2000). Seed-based correlation analysis has since been used to define brain networks such as the default mode network (DMN; Greicius et al., 2003), and to study the functional parcellation of specific brain structures, such as the cingulate cortex (Margulies et al., 2007), basal ganglia (Di Martino et al., 2008), and insula (Taylor et al., 2009).

As an alternative to seed-based analysis, where the region of interest is known, researchers have used independent component analysis (ICA), a data driven methodology to decompose the brain into spatially independent networks (McKeown et al., 1998). ICA simultaneously investigate multiple networks such as the DMN, salience, left/right executive, attention, motor, and visual networks (Greicius et al., 2004; Beckmann et al., 2005) and several successful applications have been reported in mental diseases (e.g., Greicius et al., 2004; Veer et al., 2010; Westlye et al., 2011).

The studies of functional connectivity and networks generally rely on the correlations and relative independence of low-frequency fluctuation signals of resting-state functional magnetic resonance imaging (fMRI). However, the influences of resting-state fMRI signal fluctuation amplitude on the measures of functional connectivity and networks have largely been ignored. Theoretically, the correlation coefficient should be independent of the scale of the signals. However, the reliability of fMRI signals might be associated with the level of noises as well as meaningful neuronal functions (e.g., Sirotin and Das, 2009). Therefore, the fluctuation amplitudes may indeed affect the functional connectivity and network measures.

The “noise” of the brain has been shown to characterize the developing (McIntosh et al., 2008) and the aging (Garrett et al., 2010, 2011) brain, and the variability of the noise has been shown to explain behavioral variability (for a review, see McIntosh et al., 2010). On the other hand, the resting-state fMRI is susceptible to many sources of noise such as head motion (Power et al., 2012; Van Dijk et al., 2012), respiration, and heartbeat (Birn et al., 2006, 2008; Chang et al., 2008). Data processing strategies were found to significantly affect connectivity measures (Weissenbacher et al., 2009; Saad et al., 2012), which implies that the connectivity results are still largely influenced by different sources of noise even after following careful processing procedures. Taken together, a better understanding of how the resting-state fMRI fluctuation amplitude affect functional connectivity and networks is warranted.

The fluctuations of resting-state BOLD signals are generally observed to be present between 0.01 and 0.08 Hz frequency band (Biswal et al., 1995). The amplitude of resting-state BOLD fluctuations is usually calculated in this low-frequency band, which has been termed as the amplitude of low-frequency fluctuations (ALFF, Zang et al., 2007). Higher ALFF in resting-state have been shown in regions constituting the DMN (Zang et al., 2007), suggesting that ALFF to some extent reflects neural activity. In addition, recent studies have observed an overlap between changes in regional ALFF and functional connectivity in several brain regions in stuttering (Xuan et al., 2012) and seasonal affective disorder subjects (Abou Elseoud et al., 2012). These studies suggest a relationship between ALFF and functional connectivity; however, the extent and selectivity of this association has not been investigated.

In the present study, we aimed to systematically examine the relationships between ALFF and resting-state connectivity. A large dataset of healthy old subjects were analyzed so that the inter-subject variability of ALFF and connectivity was maximized. First, spatial ICA was performed on the resting-state fMRI data to identify resting-state networks. These networks were correlated with regional ALFFs in a voxel-wise manner to examine whether the inter-subject variability of the network strengths were correlated with ALFFs. Second, functional connectivity across 160 regions of interest (ROIs) were calculated. The functional connectivity was correlated with ALFF to examine whether the local amplitude fluctuations affect the strength of connectivity. We hypothesize that the strength of connectivity of ICA and ROI based analyses would be correlated to the local ALFF. In addition, the correlations were examined across different networks and connectivity pairs to determine whether these associations were across the entire brain or specific to selective networks.

## Materials and Methods

### Resting-state MRI data

Resting-state fMRI and anatomical MRI data were obtained on a sample of old male subjects. After removing data with large head motion, 79 subjects were included with a mean age of 80.3 years (range from 65 to 92) for further analysis. A 3.0-T Siemens Magnetom Tim Trio scanner equipped with a 12-channel head coil (Erlangen, Germany) was used to acquire the MR images. All the functional and anatomical images were scanned parallel to the anterior commissure-posterior commissure line. The resting-state data were scanned for 500 s with a TR of 2.5 s, resulting in 200 images for each subject. The scanning parameters were as follows: TE = 27; acquisition matrix = 64 × 64; flip angle = 77°; slices = 43; spatial resolution = 3.44 mm × 3.44 mm × 3.40 mm. High resolution MPRAGE anatomical images were also acquired with the scanning parameters as follows: TR = 2530 ms; TE = 3.5 ms; flip angle = 7°; resolution = 1 mm × 1 mm × 1 mm (no gap).

### Data analysis

#### Preprocessing

The functional and anatomical image preprocessing were performed using SPM8 toolbox^1^ under MATLAB7.7 software^2^. The first two functional images were discarded. Then, the remaining functional images were motion corrected and coregistered to the subjects’ own anatomical images. The anatomical images were segmented using the new segmentation routine in SPM8. The deformation field maps obtained in segmentation were used to normalize all the functional images into standard Montreal Neurological Institute (MNI) space. For each voxel, the six rigid body head motion parameters, the first five eigenvectors from white matter (WM) signals, and the first five eigenvectors from cerebrospinal fluid (CSF) signals were regressed out using linear regression. The WM and CSF masks were defined for each subject using the segmented WM and CSF images thresholded at *p* > 0.99. Finally, all the functional images were spatially smoothed using a Gaussian kernel of 8 mm full width at half maximum (FWHM).

#### Calculation of ALFF

Amplitude of low-frequency fluctuations maps were calculated between 0.01 and 0.08 Hz band using Resting-State fMRI Data Analysis Toolkit V1.6 (REST; Song et al., 2011). The ALFF maps were then divided by whole brain mean ALFF values to normalize the global effects.

#### Relationships between network strength and ALFF

Spatial ICA was conducted to define intrinsic networks using the Group ICA of fMRI Toolbox (GIFT)^3^ (Calhoun et al., 2001). Twenty components were extracted. Resting-state networks were visually identified according to the literature (Biswal et al., 2010; Cole et al., 2010). These ICs were back-reconstructed to each subject using group ICA algorithm, resulting in 20 IC maps for each subject (Erhardt et al., 2011). To examine whether there was a consistent network effect across subjects, voxel-wise one-sample *t* tests was performed for each of the networks. The resulting *t* maps were thresholded at |*t*| > 3.42 (*p* < 0.001).

A voxel-matched correlation analysis was used to study the relationships between resting-state network strengths and ALFFs (similar to Mennes et al., 2010, 2011). For each voxel, network strengths of an IC were correlated with ALFFs across all subjects using Pearson’s correlation coefficient. The correlation maps were calculated separately for each of the network maps. Some voxels within an IC had negative value which reflects a negative relationship between a given voxel to the corresponding IC. Therefore, negative correlation between ALFF and negative IC strength is equivalent to positive correlation between ALFF and positive IC strength.

The resulting *r* maps were thresholded at |*r*| > 0.364 (*p* < 0.001). Because the aim of the current analysis was to show the overall correlation patterns, we did not use multiple comparison correction. However, a Monte Carlo simulation using AlphaSim^4^ indicated that a cluster exceeding 24 voxels were significant at *p* < 0.05 after a whole brain multiple comparison correction. This analysis shows that most of our large clusters reported in the results were still significant even after multiple comparison correction.

#### Relationships between functional connectivity and ALFF

Mean time series from 160 functionally defined ROIs were calculated within spherical ROIs with 8 mm radius (Dosenbach et al., 2010). These 160 ROIs were also assigned into six networks according to a modularity analysis of resting-state data (Dosenbach et al., 2010), including the cerebellar, cingulo-opercular, DMN, fronto-parietal, occipital, and sensorimotor networks (see Table A1 in Appendix for details). Then, functional connectivity matrices were calculated for each subject using Pearson’s correlation coefficient across 160 ROIs. The connectivity matrices were transformed into Fisher’s *z*. For each of the ROI, the Fisher’s *z* scores between a given ROI to other ROIs were correlated with ALFF value of the given ROI.

To identify which ROI’s local ALFF were more likely to correlate with connectivity, the correlations were thresholded at |*r*| > 0.364 (*p* < 0.001). Then, we selected the ROIs with local ALFFs that were correlated with more than 30 significant connectivity between the given ROI and other ROIs. These ROIs and the corresponding connections with other ROIs were visualized using BrainNet Viewer^5^.

## Results

### Relationships between network strength and ALFF

**Figure 1:**
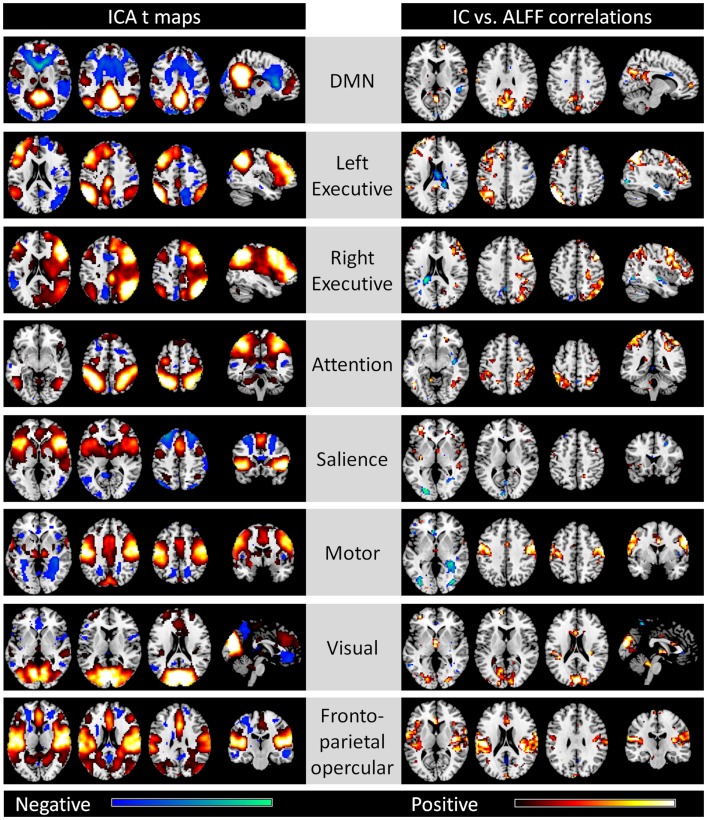
**Eight networks identified by spatial ICA that correspond to Cole et al. (2010) (left column) and voxel-wise correlations between network strengths and ALFFs (right column)**. All maps are thresholded at *p* < 0.001. For the ICA *t* maps, displayed range is absolute *t* value between 3.42 and 20, and for the correlation maps, display range is absolute *r* value between 0.364 and 0.6. Hot and cold colors encode positive and negative effects, respectively.

Out of the 20 ICs, 8 ICs were identified which corresponded to the 8 networks described by Cole et al. (2010), including the DMN, left and right executive, attention, salience, motor, visual, and fronto-parietal opercular networks (the left column of Figure 1). The voxel-matched correlations between network strengths and ALFFs for the eight networks were shown in the right column. Strong correlations between network strengths and ALFFs were generally observed within each network with less spatial extent when using the compatible statistical threshold of *p* < 0.001. For the DMN network, correlations between network strengths and ALFFs were observed in the posterior cingulate cortex/precuneus, medial prefrontal cortex (MPFC), and the right inferior parietal lobule/superior temporal gyrus. Within the left and right executive network, correlations were identified in the left and right dorsolateral prefrontal cortex and superior parietal lobule, respectively. The attention network demonstrated correlations within the bilateral superior parietal lobule and middle temporal gyrus. In the salience network, although clusters of high correlations were unapparent, there were small clusters within the bilateral insula and inferior frontal gyrus. For the motor network, correlations were observed in the bilateral sensorimotor cortex and supplementary motor area. In the visual network, correlations were identified primarily in the visual cortex. Lastly, correlations were observed in the bilateral insula/inferior frontal gyrus, and cingulate cortex within the fronto-parietal opercular network.

**Figure 2:**
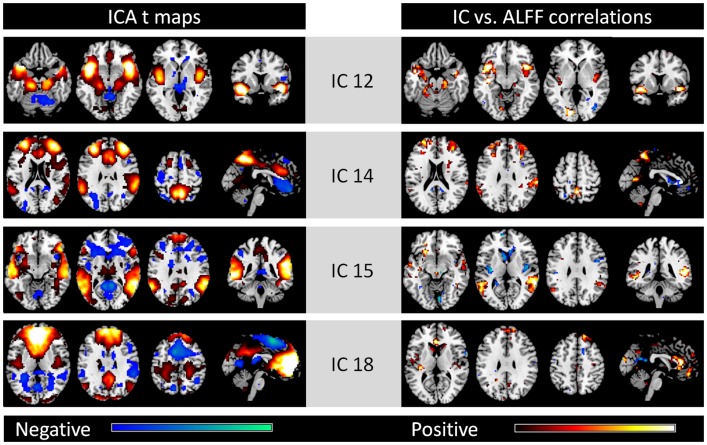
**Other four networks identified by spatial ICA (left column) and voxel-wise correlations between network strengths and ALFFs (right column)**. All maps are thresholded at *p* < 0.001. For the ICA *t* maps, display range is absolute *t* value between 3.42 and 20, and for the correlation maps, display range is absolute *r* value between 0.364 and 0.6. Hot and cold colors encode positive and negative effects, respectively.

In addition to the eight ICs, four other ICs were considered to be meaningful brain networks (the left column of Figure 2). IC 12 was mainly comprised of the bilateral insula, bilateral anterior temporal lobe, bilateral hippocampal gyrus, and bilateral amygdala. IC 14 included regions within the bilateral superior frontal gyrus, medial frontal gyrus, and bilateral inferior parietal lobe, whereas IC 15 was mostly within the bilateral temporal lobe. IC 18 was mainly located in the MPFC, anterior cingulate cortex, and posterior cingulate cortex. High correlation between IC strengths and regional ALFFs were also observed in these regions of each network, respectively (right column).

**Figure A1:**
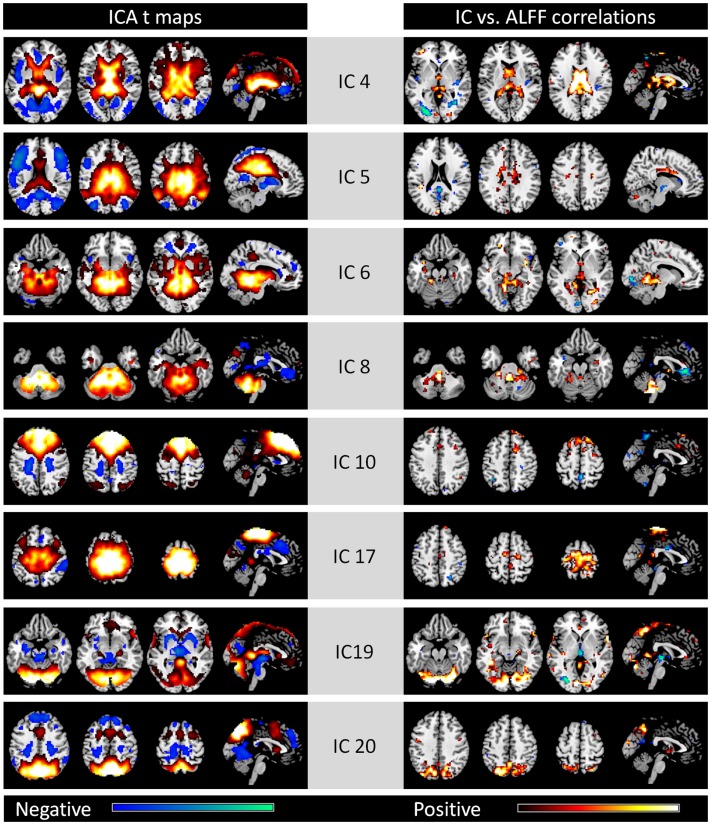
**Eight noise networks identified by spatial ICA (left column) and voxel-wise correlations between network strengths and ALFFs (right column)**. All maps are thresholded at *p* < 0.001. For the ICA *t* maps, display range is absolute *t* value between 3.42 and 20, and for the correlation maps, display range is absolute *r* value between 0.364 and 0.6. Hot and cold colors encode positive and negative effects, respectively.

We classified the remaining eight ICs as components related to noise. Voxels with high values within these ICs were mainly located in the CSF, WM, or large vessels (see Figure A1 in Appendix). We also observed high correlations between these IC strengths and ALFFs.

### Relationships between functional connectivity and ALFF

**Figure 3:**
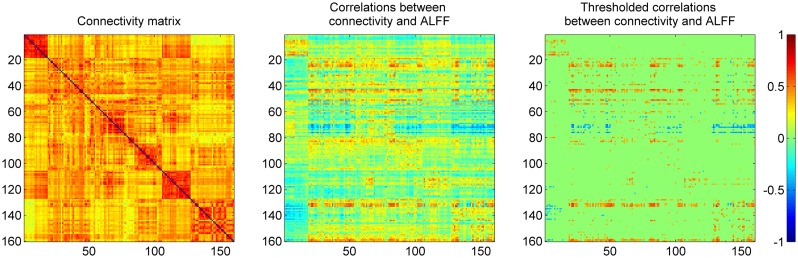
**Mean correlation matrix of resting-state connectivity across 160 ROIs (left), and their relationships to regional ALFF (middle and right)**. Each row of the middle panel revealed correlations between ALFF in one ROI and connectivity between this ROI to all the other ROIs. The thresholded correlations between ALFF and connectivity were shown in the right panel (|*r*| > 0.364, i.e., *p* < 0.001).

The mean connectivity matrix across 160 ROIs is illustrated in the left panel of Figure 3. Even with strong connectivity coefficient values, we observed higher connectivity within each network compared with between networks. These ROIs were sorted by their six network affiliations (see Table A1 in Appendix), and high correlation values within the networks are evident as subsquares along the mean connectivity matrix diagonal, for example the cerebellar network (ROI 1–18), DMN (ROI 51–84), fronto-parietal network (ROI 85–105), occipital network (ROI 106–127), and sensorimotor network (ROI 128–160). However, we did not observe strong within network connectivity of the cingulo-opercular network (ROI 19–50).

The correlation between the ALFF of a given ROI and the connectivity between the given ROI with other ROIs are illustrated in the middle panel of Figure 3. The matrix was thresholded (|*r*| > 0.364, i.e., *p* < 0.001) to determine which correlation between the ALFF of a given ROI and its connectivity were statistically significant. The right panel of Figure 3 demonstrates that the matrix was asymmetrical with respect to the diagonal which suggests that ALFFs of both ROIs within a pair affect functional connectivity differently. It also demonstrates that ALFF of specific ROIs were more likely to influence the connectivity between these specific ROIs with other ROIs.

**Figure 4:**
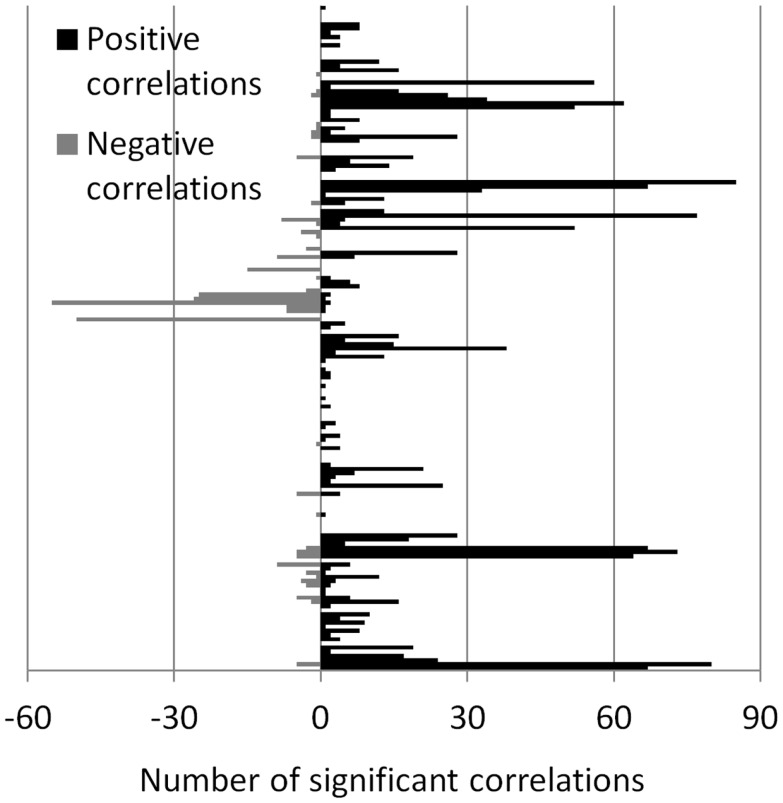
**Number of significant correlations between ALFF and connectivity for each ROI**. The number of positive and negative correlations were shown separately, and displayed in positive and negative directions, respectively.

The number of positive and negative correlations correlated with the local ALFF was tabulated (Figure 4) to identify the regions where the local ALFF were more likely to affect connectivity. We set an arbitrary threshold of *n* > 30 to identify these regions (see Table 1). Fifteen ROIs revealed more than 30 connections that were positively correlated with ALFFs from the corresponding ROIs, while two ROIs revealed more than 30 connections that were negatively correlated with ALFFs from the corresponding ROIs.

**Table 1:** **ROIs that have more than 30 connections that are correlated with the corresponding regional ALFF**.

Label	ROI #	Network	MNI coordinates		
			*x*	*y*	*z*
**POSITIVE EFFECTS**					
ACC	19	Cingulo-opercular	−2	30	27
aPFC	23	Cingulo-opercular	27	49	26
Basal ganglia	24	Cingulo-opercular	14	6	7
Basal ganglia	25	Cingulo-opercular	−20	6	7
Thalamus	43	Cingulo-opercular	−12	−3	13
Thalamus	44	Cingulo-opercular	−12	−12	6
Thalamus	45	Cingulo-opercular	11	−12	6
ACC	51	Default	9	39	20
aPFC	54	Default	−25	51	27
vmPFC	83	Default	−11	45	17
Mid insula	131	Sensorimotor	−42	−3	11
Mid insula	132	Sensorimotor	−36	−12	15
Mid insula	133	Sensorimotor	33	−12	16
vFC	159	Sensorimotor	43	1	12
vFC	160	Sensorimotor	−55	7	23
**NEGATIVE EFFECTS**					
Precuneus	72	Default	5	−50	33
Precuneus	76	Default	−6	−56	29

**Figure 5:**
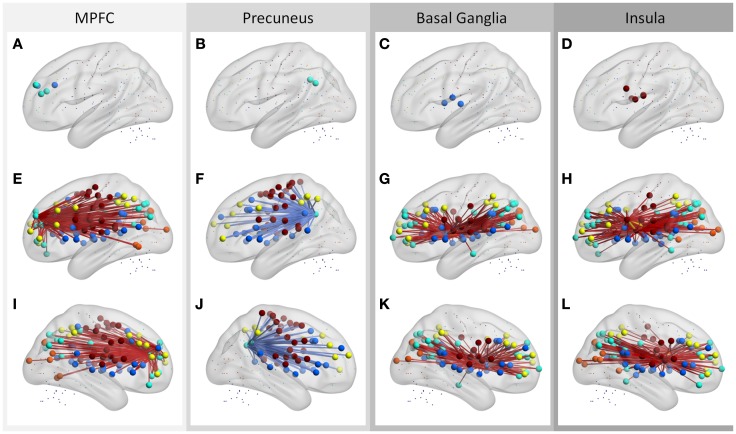
**Regions that have more than 30 connections correlated with regional ALFF (A–D), and their associated connections (E–L)**. The ROIs are stratified into four sets according to their affiliated networks and connectivity behavior (one for each column). Middle and bottom rows showed left and right lateral views, respectively. Hot and cold colors of connections indicate positive and negative correlations. Color codes of the ROIs: blue, cingulo-opercular network; cyan, DMN; yellow, fronto-parietal network; orange, occipital network; brown, sensorimotor network.

These ROIs were categorized into four groups based on their spatial approximations and affiliated networks. The first group of ROIs were located in the MPFC and anterior cingulate cortex (ACC) (Figure 5A). These five ROIs were either a part of the DMN or the cingulo-opercular network as described by Dosenbach et al. (2010); however, these nearby ROIs exhibited similar correlation patterns. The connectivity between the five ROIs with other DMN, fronto-parietal, cingulo-opercular, sensorimotor, and occipital regions demonstrated positive correlations with local ALFF (Figures 5E,I). The second set of ROIs were located in the precuneus (Figure 5B), and the connectivity of these ROIs to cingulo-opercular, fronto-parietal, and sensorimotor regions were negatively correlated with local ALFF (Figures 5F,J). The third set was comprised of five ROIs in the bilateral putamen, caudate, and thalamus (Figure 5C), and their connectivity to the DMN, fronto-parietal, cingulo-opercular, and occipital regions revealed positive correlations with local ALFF (Figures 5G,K). The fourth group of ROIs were located at the bilateral insula and ventral fontal regions (Figure 5D), and their connectivity to the DMN, fronto-parietal, cingulo-opercular, and occipital regions revealed positive correlations with local ALFF (Figures 5H,L).

## Discussion

The current analysis demonstrates that the network strengths as measured by ICA were selectively correlated with ALFFs within the corresponding network. The network strength measured by ICA reflects the extent that a particular voxel correlates with the whole IC. Thus, the correlations between ALFFs and network strengths imply that the within network connectivity are correlated with the local fluctuation amplitudes. The relationship between ICA and ALFF were replicated by directly correlating ALFFs with connectivity measured via correlations. Within each network, ALFFs were positively correlated with the connectivity and were demonstrated as squares within each network nearby the diagonal of the matrix (see the right panel of Figure 3, e.g., cerebellar and sensorimotor networks). Interestingly, the correlation between ALFFs and connectivity were not restricted to within network but extends to between network connectivity. The functional connectivity of regions, particularly the MPFC, ACC, precuneus, basal ganglia, thalamus, and insula, with other regions were widely spread in the whole brain and suggest a special role of these regions in functional connectivity pattern.

The association between local fluctuations and connectivity may simply reflect that the BOLD signals are more reliable with less noise. However, given that the correlations are not uniform across the whole brain and that selective correlations are between specific regional ALFFs and connectivity, these associations may suggest functional significance. One possible explanation is that these selective regions may be involved in transmitting information to various brain regions, such that the greater the neural activity results in larger regional amplitude of fluctuations, and greater connectivity between these regions to other regions. In addition, the variances of ALFF may reflect different levels of neurotransmitters that give rise to functional connectivity variances. The later notion can be tested by combining resting-state fMRI with magnetic resonance spectroscopy (MRS) or positron emission tomography (PET) (Horn et al., 2010; Hahn et al., 2011; Cole et al., 2012; Kapogiannis et al., 2013).

Alternatively, it is also possible that these regions are more likely to be impacted by physiological noise. Even though ALFF is considered to be a measure of amplitude of neural activity, our recent studies have shown that ALFF is highly correlated with neurovascular response of breath holding task (Biswal et al., 2007; Di et al., 2013). In addition, the regions that demonstrate high correlations between ALFF and connectivity were also the regions that were more likely to be affected by physiological noise due to the adjacent large vessels, including the MPFC and precuneus, and insula (Di et al., 2013). These physiological noises may also influence functional connectivity (Birn et al., 2006, 2008; Chang et al., 2008), and therefore, reflect the common sources of physiological noise that affects both measures. Consistent with this notion, the ICs that reflected physiological noises exhibited high correlations in the regions located in the CSF, WM, and large vessels (see Figure A1 in Appendix). However, for the ICs that reflect meaningful neural networks, the correlations between ALFF and network strength may reflect both neural and noise contributions.

The first two sets of ROIs exhibiting correlations between connectivity and ALFF were within the DMN, including the MPFC/ACC regions, and precuneus. These regions are also defined as the structural core of the human brain that has the most anatomical connections to other brain regions (Hagmann et al., 2008). Most interestingly, the correlations between regional ALFF and connectivity showed reversed relationships between the prefrontal regions and precuneus. The connectivity between the two ROIs in precuneus with other regions was negatively correlated with the precuneus ALFF. These brain regions were task positive networks such as the sensorimotor, fronto-parietal, and cingulo-opercular networks. The connectivity between DMN and task positive networks is generally negative (Fox et al., 2005), which suggests that greater regional ALFF is associated with greater negative connectivity between DMN and task positive networks. In addition, we did not apply global scaling on the current dataset in order to prevent artificial negative correlations and no negative connectivity was observed. Thus, the negative relationship between connectivity (DMN and task positive networks) and ALFF is not due to preprocessing of the data. In contrast, the connectivity between MPFC/ACC ROIs and other regions revealed positive correlations with ALFF. MPFC/ACC ROIs were correlated with regions within other areas of the DMN, and with regions of the fronto-parietal, sensorimotor, cingulo-opercular, and occipital networks. These different correlation pattern suggests that the modulation of connectivity may involve different underlying mechanisms, e.g., via excitatory and inhibitory neurotransmitter modulations. Glutamate concentration, which reflects excitatory mechanisms in the ACC (Horn et al., 2010) and posteromedial cortex (Kapogiannis et al., 2013) has been shown to positively modulate the resting-state functional connectivity. In contrast, GABA concentration, which reflects inhibitory mechanisms, in the posteromedial cortex has been shown to negatively correlate with the resting-state functional connectivity. However, the links between the amplitude of fluctuations and neurotransmitter concentrations is still largely unknown, thus require further studies.

The other two sets of ROIs include the basal ganglia, thalamus, insula, and adjacent sensorimotor regions. Previous studies have demonstrated a widely spread functional connectivity of these regions to other brain regions (e.g., Di Martino et al., 2008; Taylor et al., 2009; Cauda et al., 2011; Tang et al., 2011). The positive correlations between connectivity and ALFF suggest a special role of these regions in functional connectivity pattern.

A practical implication of the present result is that when studying resting-state functional connectivity or networks, ALFF may be a potential confounding variable that needs to be taken into account. ALFF has been widely used to study the “baseline” activity of a wide spectrum of psychological states and mental diseases, for example aging (Biswal et al., 2010; Yan et al., 2011), schizophrenia (Hoptman et al., 2010; Huang et al., 2010), and attention deficit hyperactivity disorder (ADHD; Zang et al., 2007). Distributed differences of ALFF have been observed to be associated with different pathologies and mental states. On the other hand, increasingly studies have been conducted to investigate brain functional connectivity alterations in mental diseases using both seed-based correlation and spatial ICA (e.g., Greicius et al., 2004; Castellanos et al., 2008; Veer et al., 2010; Westlye et al., 2011). Although these differences are presumed to reflect the group differences in resting-state connectivity and networks, ALFF was not controlled by previous studies. Therefore, the underlying group differences in functional connectivity that have been reported by previous studies may be due to the unrestrained ALFF. By including ALFF as covariance, Abou Elseoud et al. (2012) demonstrated increased connectivity, but less number of voxels in the visual network. Thus, the current result and that of Abou Elseoud et al. (2012) raises a concern regarding ALFF as a potential confound when study functional connectivity and network. More specifically, our data suggests that one should be cautious when interpreting seed-based correlations of regions that are more likely to be affected by ALFF, such as the precuneus, MPFC, basal ganglia, thalamus, and insula.

The present study only analyzed a sample of old individuals because old subjects typically demonstrate larger variance of functional connectivity and the associations of functional connectivity with ALFF may be easier to identify. Even though we believe that the current results will also hold for younger individuals, further studies investigating younger individuals is needed to determine whether the relationship between local fluctuation amplitudes and functional connectivity generalizes to young population.

## Conflict of Interest Statement

The authors declare that the research was conducted in the absence of any commercial or financial relationships that could be construed as a potential conflict of interest.

## Footnotes

^1^http://www.fil.ion.ucl.ac.uk/spm/

^2^http://www.mathworks.com/

^3^http://icatb.sourceforge.net/

^4^http://afni.nimh.nih.gov/pub/dist/doc/manual/AlphaSim.pdf

^5^http://www.nitrc.org/projects/bnv/

## Appendix

**Table A1:** **One hundred sixty functionally defined ROIs used in the current study**.

ROI #	*x*	*y*	*z*	Label	Network
1	−34	−67	−29	Inf cerebellum	Cerebellum
2	32	−61	−31	Inf cerebellum	Cerebellum
3	−25	−60	−34	Inf cerebellum	Cerebellum
4	−37	−54	−37	Inf cerebellum	Cerebellum
5	18	−81	−33	Inf cerebellum	Cerebellum
6	−6	−79	−33	Inf cerebellum	Cerebellum
7	−21	−79	−33	Inf cerebellum	Cerebellum
8	33	−73	−30	Inf cerebellum	Cerebellum
9	−24	−54	−21	Lat cerebellum	Cerebellum
10	21	−64	−22	Lat cerebellum	Cerebellum
11	−28	−44	−25	Lat cerebellum	Cerebellum
12	−34	−57	−24	Lat cerebellum	Cerebellum
13	14	−75	−21	Med cerebellum	Cerebellum
14	1	−66	−24	Med cerebellum	Cerebellum
15	−6	−60	−15	Med cerebellum	Cerebellum
16	−16	−64	−21	Med cerebellum	Cerebellum
17	5	−75	−11	Med cerebellum	Cerebellum
18	−11	−72	−14	Med cerebellum	Cerebellum
19	−2	30	27	ACC	Cingulo-opercular
20	−41	−47	29	Angular gyrus	Cingulo-opercular
21	38	21	−1	Ant insula	Cingulo-opercular
22	−36	18	2	Ant insula	Cingulo-opercular
23	27	49	26	aPFC	Cingulo-opercular
24	14	6	7	Basal ganglia	Cingulo-opercular
25	−20	6	7	Basal ganglia	Cingulo-opercular
26	−6	17	34	Basal ganglia	Cingulo-opercular
27	11	−24	2	Basal ganglia	Cingulo-opercular
28	9	20	34	dACC	Cingulo-opercular
29	54	−31	−18	Fusiform	Cingulo-opercular
30	0	15	45	mFC	Cingulo-opercular
31	37	−2	−3	Mid insula	Cingulo-opercular
32	−30	−14	1	Mid insula	Cingulo-opercular
33	32	−12	2	Mid insula	Cingulo-opercular
34	−55	−44	30	Parietal	Cingulo-opercular
35	58	−41	20	Parietal	Cingulo-opercular
36	−4	−31	−4	Post cingulate	Cingulo-opercular
37	−30	−28	9	Post insula	Cingulo-opercular
38	8	−40	50	Precuneus	Cingulo-opercular
39	42	−46	21	Sup temporal	Cingulo-opercular
40	43	−43	8	Temporal	Cingulo-opercular
41	−59	−47	11	Temporal	Cingulo-opercular
42	51	−30	5	Temporal	Cingulo-opercular
43	−12	−3	13	Thalamus	Cingulo-opercular
44	−12	−12	6	Thalamus	Cingulo-opercular
45	11	−12	6	Thalamus	Cingulo-opercular
46	−52	−63	15	TPJ	Cingulo-opercular
47	−46	10	14	vFC	Cingulo-opercular
48	−48	6	1	vFC	Cingulo-opercular
49	51	23	8	vFC	Cingulo-opercular
50	34	32	7	vPFC	Cingulo-opercular
51	9	39	20	ACC	Default
52	−48	−63	35	Angular gyrus	Default
53	51	−59	34	Angular gyrus	Default
54	−25	51	27	aPFC	Default
55	28	−37	−15	Fusiform	Default
56	−59	−25	−15	Inf temporal	Default
57	−61	−41	−2	Inf temporal	Default
58	52	−15	−13	Inf temporal	Default
59	−36	−69	40	IPS	Default
60	0	51	32	mPFC	Default
61	45	−72	29	Occipital	Default
62	−9	−72	41	Occipital	Default
63	−42	−76	26	Occipital	Default
64	−28	−42	−11	Occipital	Default
65	−2	−75	32	Occipital	Default
66	10	−55	17	Post cingulate	Default
67	−11	−58	17	Post cingulate	Default
68	−8	−41	3	Post cingulate	Default
69	1	−26	31	Post cingulate	Default
70	−5	−52	17	Post cingulate	Default
71	−5	−43	25	Post cingulate	Default
72	5	−50	33	Precuneus	Default
73	11	−68	42	Precuneus	Default
74	9	−43	25	Precuneus	Default
75	−3	−38	45	Precuneus	Default
76	−6	−56	29	Precuneus	Default
77	23	33	47	Sup frontal	Default
78	−16	29	54	Sup frontal	Default
79	46	39	−15	vlPFC	Default
80	6	64	3	vmPFC	Default
81	−6	50	−1	vmPFC	Default
82	9	51	16	vmPFC	Default
83	−11	45	17	vmPFC	Default
84	8	42	−5	vmPFC	Default
85	−1	28	40	ACC	Fronto-parietal
86	29	57	18	aPFC	Fronto-parietal
87	−29	57	10	aPFC	Fronto-parietal
88	−42	7	36	dFC	Fronto-parietal
89	40	17	40	dFC	Fronto-parietal
90	44	8	34	dFC	Fronto-parietal
91	40	36	29	dlPFC	Fronto-parietal
92	46	28	31	dlPFC	Fronto-parietal
93	−44	27	33	dlPFC	Fronto-parietal
94	−48	−47	49	IPL	Fronto-parietal
95	−41	−40	42	IPL	Fronto-parietal
96	−53	−50	39	IPL	Fronto-parietal
97	44	−52	47	IPL	Fronto-parietal
98	54	−44	43	IPL	Fronto-parietal
99	−32	−58	46	IPS	Fronto-parietal
100	32	−59	41	IPS	Fronto-parietal
101	−35	−46	48	Post parietal	Fronto-parietal
102	42	48	−3	Vent aPFC	Fronto-parietal
103	−43	47	2	Vent aPFC	Fronto-parietal
104	39	42	16	vlPFC	Fronto-parietal
105	−52	28	17	vPFC	Fronto-parietal
106	−44	−63	−7	Occipital	Occipital
107	17	−68	20	Occipital	Occipital
108	36	−60	−8	Occipital	Occipital
109	−34	−60	−5	Occipital	Occipital
110	39	−71	13	Occipital	Occipital
111	19	−66	−1	Occipital	Occipital
112	−16	−76	33	Occipital	Occipital
113	9	−76	14	Occipital	Occipital
114	15	−77	32	Occipital	Occipital
115	29	−73	29	Occipital	Occipital
116	−29	−75	28	Occipital	Occipital
117	20	−78	−2	Occipital	Occipital
118	−18	−50	1	Occipital	Occipital
119	−29	−88	8	Post occipital	Occipital
120	13	−91	2	Post occipital	Occipital
121	27	−91	2	Post occipital	Occipital
122	−4	−94	12	Post occipital	Occipital
123	−5	−80	9	Post occipital	Occipital
124	29	−81	14	Post occipital	Occipital
125	33	−81	−2	Post occipital	Occipital
126	−37	−83	−2	Post occipital	Occipital
127	46	−62	5	Temporal	Occipital
128	60	8	34	dFC	Sensorimotor
129	58	11	14	Frontal	Sensorimotor
130	53	−3	32	Frontal	Sensorimotor
131	−42	−3	11	Mid insula	Sensorimotor
132	−36	−12	15	Mid insula	Sensorimotor
133	33	−12	16	Mid insula	Sensorimotor
134	−26	−8	54	Parietal	Sensorimotor
135	−47	−18	50	Parietal	Sensorimotor
136	−38	−15	59	Parietal	Sensorimotor
137	46	−20	45	Parietal	Sensorimotor
138	−55	−22	38	Parietal	Sensorimotor
139	−38	−27	60	Parietal	Sensorimotor
140	−24	−30	64	Parietal	Sensorimotor
141	41	−23	55	Parietal	Sensorimotor
142	18	−27	62	Parietal	Sensorimotor
143	−47	−12	36	Parietal	Sensorimotor
144	42	−24	17	Post insula	Sensorimotor
145	−41	−31	48	Post parietal	Sensorimotor
146	10	5	51	Pre-SMA	Sensorimotor
147	−54	−22	22	Precentral gyrus	Sensorimotor
148	−54	−9	23	Precentral gyrus	Sensorimotor
149	44	−11	38	Precentral gyrus	Sensorimotor
150	−44	−6	49	Precentral gyrus	Sensorimotor
151	46	−8	24	Precentral gyrus	Sensorimotor
152	58	−3	17	Precentral gyrus	Sensorimotor
153	0	−1	52	SMA	Sensorimotor
154	34	−39	65	Sup parietal	Sensorimotor
155	−53	−37	13	Temporal	Sensorimotor
156	−41	−37	16	Temporal	Sensorimotor
157	59	−13	8	Temporal	Sensorimotor
158	−54	−22	9	Temporal	Sensorimotor
159	43	1	12	vFC	Sensorimotor
160	−55	7	23	vFC	Sensorimotor

*The ROIs were obtained from Dosenbach et al. (2010), and sorted by their affiliating networks. The *x*, *y*, and *z* coordinates were given in MNI space*.
